# Supplementary material for: Early Changes in LIPI Score Predict Immune-Related Adverse Events: A Propensity Score Matched Analysis in Advanced Non-Small Cell Lung Cancer Patients on Immune Checkpoint Blockade
Source: Cancers (Basel). 2024 Jan 20;16(2):453. doi: 10.3390/cancers16020453 (PMC10814049; doi:10.3390/cancers16020453)
Supplement: Supplementary file 1 [file cancers-16-00453-s001.zip › cancers-2822620-supplementary.pdf]

SUPPLEMENTARY MATERIAL

Article

Early Changes in LIPI Score Predict Immune-Related Adverse Events: A Propensity Score Matched Analysis in Advanced Non-Small Cell Lung Cancer Patients on Immune Checkpoint Blockade

Fabrizio Nelli [1,*], Agnese Fabbri [1], Antonella Virtuoso [1], Diana Giannarelli [2], Julio Rodrigo Giron Berrios [1], Eleonora Marrucci [1], Cristina Fiore [1] and Enzo Maria Ruggeri [1]

[1] Medical Oncology Unit, Central Hospital of Belcolle, Department of Oncology and Hematology, Strada Sammartinese snc, 01100 Viterbo, Italy
[2] Biostatistics Unit, Scientific Directorate, Fondazione Policlinico Universitario A. Gemelli, Istituto di Ricovero e Cura a Carattere Scientifico (IRCCS), 00168 Rome, Italy
* Correspondence: fabrizio.nelli@asl.vt.it; Tel./Fax: +39-0761339055

Supplementary Table S1. Patient characteristics.

Characteristic	General population					PSM population				
	All patients (N=345)	LIPI-0 (N=115)	LIPI-1 (N=118)	LIPI-2 (N=112)	P value	All patients (N=249)	LIPI-0 (N=83)	LIPI-1 (N=83)	LIPI-2 (N=83)	P value
Age										
- Mean (SD), years	71.0 (8.72)	69.0 (8.70)	71.0 (8.76)	71.5 (8.76)	0.205	71.0 (8.86)	69.0 (9.44)	71 (8.36)	71.0 (8.73)	0.286
- ≥70 years	187 (54.2%)	56 (48.7%)	70 (59.3%)	61 (54.5%)	0.265	139 (55.8%)	40 (48.2%)	55 (66.3%)	44 (53.0%)	0.052
Sex					0.734					0.706
- Female	102 (29.6%)	34 (29.6%)	35 (29.7%)	33 (29.5%)		75 (30.1%)	26 (31.3%)	25 (30.1%)	24 (28.9%)	
- Male	243 (70.4%)	81 (70.4%)	83 (70.3%)	79 (70.5%)		174 (69.9%)	57 (68.7%)	58 (69.9%)	59 (71.1%)	
ECOG PS					<0.001					0.073
- 0 or 1	268 (77.7%)	93 (80.9%)	102 (86.4%)	73 (65.2%)		200 (80.3%)	71 (85.5%)	69 (83.1%)	60 (72.3%)	
- 2	77 (22.3%)	22 (19.1%)	16 (13.6%)	39 (34.8%)		49 (19.7%)	12 (14.5%)	14 (16.9%)	23 (27.7%)	
Histologic subtype					0.423					0.244
- Nonsquamous	261 (75.7%)	87 (75.7%)	85 (72.0%)	89 (79.5%)		185 (74.3%)	60 (72.3%)	58 (69.9%)	67 (80.7%)	
- Squamous	84 (24.3%)	28 (24.3%)	33 (28.0%)	23 (20.5%)		64 (25.7%)	23 (27.7%)	25 (30.1%)	16 (19.3%)	
No. of metastatic sites					0.139					0.136
- ≤2	184 (53.3%)	70 (60.9%)	58 (49.2%)	56 (50.0%)		122 (49.0%)	48 (57.8%)	36 (43.4%)	38 (45.8%)	
- >2	161 (46.7%)	45 (39.1%)	60 (50.8%)	56 (50.0%)		127 (51.0%)	37 (42.2%)	47 (56.6%)	45 (54.2%)	
Bone metastases	90 (26.1%)	22 (19.1%)	29 (24.6%)	39 (34.8%)	0.024	69 (27.7%)	19 (22.9%)	22 (26.5%)	28 (33.7%)	0.283
Brain metastases	75 (21.7%)	26 (22.6%)	22 (18.6%)	27 (24.1%)	0.581	52 (20.9%)	17 (20.5%)	14 (16.9%)	21 (25.3%)	0.407
Liver metastases	38 (11.0%)	9 (7.8%)	16 (12.6%)	13 (11.6%)	0.366	31 (12.4%)	8 (9.6%)	13 (15.7%)	10 (12.0%)	0.497
PD-L1 TPS					0.115					0.057
-<1%	166 (48.1%)	46 (40.0%)	65 (55.1%)	55 (49.1%)		131 (52.6%)	37 (44.6%)	51 (61.4%)	43 (51.8%)	
- ≥1% and ≤49%	107 (31.0%)	40 (34.8%)	36 (30.5%)	31 (27.7%)		77 (30.9%)	33 (39.7%)	23 (27.7%)	21 (25.3%)	
- ≥50%	72 (20.9%)	29 (25.2%)	17 (14.4%)	26 (23.2%)		41 (16.5%)	13 (15.7%)	9 (10.8%)	19 (22.9%)	
BMI										
- Mean (SD), (kg/m2)	24.9 (4.80)	25.9 (5.05)	24.8 (4.69)	24.3 (4.60)	0.287	24.8 (4.73)	25.1 (4.82)	24.8 (4.77)	24.1 (4.62)	0.518
- ≥25	172 (49.8%)	64 (55.6%)	57 (48.3%)	51 (45.5%)	0.287	119 (47.8%)	42 (50.6%)	40 (48.2%)	37 (44.6%)	0.737
Smoking habits					0.081					0.115

- never<br>- ever	37 (10.7%)<br>308 (89.3%)	7 (6.1%)<br>108 (93.9%)	18 (15.3%)<br>100 (84.7%)	12 (10.7%)<br>100 (89.3%)		30 (12.0%)<br>219 (88.0%)	7 (8.4%)<br>76 (91.6%)	15 (18.1%)<br>68 (81.9%)	8 (9.6%)<br>75 (90.4%)	
Previous thoracic RT	45 (13.0%)	18 (15.6%)	15 (12.7%)	12 (10.7%)	0.539	29 (11.6%)	14 (16.9%)	7 (8.4%)	8 (9.6%)	0.187
Autoimmune disease	13 (3.8%)	4 (3.5%)	5 (4.2%)	4 (3.6%)	0.946	6 (2.4%)	1 (1.2%)	3 (3.6%)	2 (2.4%)	0.599
Corticosteroids [a]	137 (39.7%)	34 (29.6%)	51 (43.2%)	52 (46.4%)	0.022	110 (44.2%)	28 (33.7%)	41 (49.4%)	41 (49.4%)	0.064
APAP [b]	123 (35.6%)	33 (28.7%)	42 (35.6%)	48 (42.6%)	0.084	89 (35.7%)	26 (31.3%)	33 (39.7%)	30 (36.1%)	0.524
Systemic antibiotics [c]	65 (18.8%)	21 (18.3%)	24 (20.3%)	20 (17.6%)	0.874	54 (21.7%)	15 (18.1%)	19 (22.9%)	20 (24.1%)	0.609
PPI	121 (35.1%)	37 (32.2%)	44 (37.3%)	40 (35.7%)	0.705	92 (36.9%)	30 (36.1%)	33 (39.7%)	29 (34.9%)	0.799
Statins	111 (32.2%)	37 (32.2%)	39 (33.0%)	35 (31.2%)	0.958	86 (34.5%)	27 (32.5%)	29 (34.9%)	30 (36.1%)	0.883
Fibrates	63 (18.3%)	27 (23.5%)	18 (15.3%)	18 (16.1%)	0.205	42 (16.9%)	14 (16.9%)	12 (14.5%)	16 (19.3%)	0.709
NSAIDs or ASA	81 (23.5%)	25 (21.7%)	25 (21.2%)	31 (27.7%)	0.441	54 (21.7%)	19 (22.9%)	15 (18.1%)	20 (24.1%)	0.609
Beta-blockers	85 (24.6%)	35 (30.4%)	29 (24.6%)	21 (18.7%)	0.124	60 (24.1%)	22 (26.5%)	19 (22.9%)	19 (22.9%)	0.821
ACEi or ARBs	108 (31.3%)	33 (28.7%)	35 (29.7%)	40 (35.7%)	0.467	81 (32.5%)	27 (32.5%)	25 (30.1%)	29 (34.9%)	0.803
Metformin	87 (25.2%)	28 (24.3%)	34 (28.8%)	25 (22.3%)	0.508	72 (28.9%)	21 (25.3%)	28 (33.7%)	23 (27.7%)	0.468
Oral or transdermal opioids	126 (36.5%)	34 (29.6%)	50 (42.4%)	42 (37.5%)	0.123	106 (42.6%)	30 (36.1%)	42 (50.6%)	34 (40.9%)	0.159
Treatment setting<br>- First-line<br>- Second-line	 165 (47.8%)<br>180 (52.2%)	 69 (60.0%)<br>46 (40.0%)	 54 (45.8%)<br>64 (54.2%)	 42 (37.5%)<br>70 (62.5%)	0.003	 121 (48.6%)<br>128 (51.4%)	 43 (51.8%)<br>40 (48.2%)	 36 (43.4%)<br>47 (56.6%)	 42 (50.6%)<br>41 (49.4%)	0.501
First-line chemotherapy<br>- Pemetrexed-based<br>- Paclitaxel-based	 94 (27.2%)<br>18 (5.2%)	 37 (32.2%)<br>7 (6.1%)	 37 (31.4%)<br>10 (8.5%)	 20 (17.8%)<br>1 (0.9%)	0.005	 67 (26.9%)<br>14 (5.6%)	 27 (32.5%)<br>5 (6.0%)	 20 (24.1%)<br>8 (9.6%)	 20 (24.1%)<br>1 (1.2%)	0.907
ICI<br>- Nivolumab<br>- Pembrolizumab<br>- Atezolizumab	 116 (33.6%)<br>213 (61.7%)<br>16 (4.6%)	 34 (29.6%)<br>78 (67.8%)<br>3 (2.6%)	 40 (33.9%)<br>74 (62.7%)<br>4 (3.4%)	 42 (37.5%)<br>61 (54.5%)<br>9 (8.0%)	0.139	 79 (31.7%)<br>159 (63.9%)<br>11 (4.4%)	 27 (32.5%)<br>53 (63.8%)<br>3 (3.6%)	 28 (33.7%)<br>52 (62.6%)<br>3 (3.6%)	 24 (28.9%)<br>54 (65.1%)<br>5 (6.0%)	0.895

PSM, propensity score matching; LIPI, lung immune prognostic index; SD, standard deviation; ECOG PS, Eastern Cooperative Oncology Group Performance Status; PD-L1 TPS, programmed cell death ligand-1 tumor proportion score; BMI, body mass index; RT, radiotherapy; APAP,

acetaminophen; PPI, proton pump inhibitors; NSAIDs, nonsteroidal anti-inflammatory drugs; ASA, acetylsalicylic acid; ACEi, angiotensin-converting enzyme inhibitors; ARBs, angiotensin II type 2 receptor blockers; ICI, immune checkpoint inhibitor.

[a] Corticosteroids indicate intake of prednisone equivalent ≥10 mg daily for at least 5 days before 14 days prior to the start of treatment (excluding premedication for chemotherapy); [b] APAP indicates a therapeutic intake of at least 1000 mg per day for more than 24 hours during the 30 days prior to the start of treatment; [c] systemic antibiotics indicate a therapeutic intake in the 30 days prior to the start of treatment.

Supplementary Table S2. Multivariate analysis of LDH levels by predefined clinical variables.

Covariate	LDH level at baseline (log)		LDH level after two cycles of treatment (log)	
	Beta (95% CI)	P value	Beta (95% CI)	P value
Age<br>- ≥70 years vs. <70 years	-0.02 (-0.06 to 0.01)	0.198	-0.02 (-0.06 to 0.01)	0.189
Sex<br>- Male vs. female	0.01 (-0.04 to 0.05)	0.879	0.01 (-0.04 to 0.05)	0.845
ECOG PS<br>- 2 vs. 0 or 1	0.04 (-0.01 to 0.09)	0.093	0.04 (-0.01 to 0.09)	0.068
Histologic subtype<br>- Squamous vs. nonsquamous	0.03 (-0.08 to 0.01)	0.124	-0.01 (-0.06 to 0.03)	0.462
Number of metastatic sites<br>- >2 vs. ≤2	-0.01 (-0.07 to 0.04)	0.627	0.01 (-0.05 to 0.05)	0.996
Bone metastases<br>- Any vs. not present	0.03 (-0.01 to 0.08)	0.213	0.02 (-0.02 to 0.39)	0.399
Brain metastases<br>- Any vs. not present	0.04 (-0.01 to 0.09)	0.135	0.02 (-0.02 to 0.07)	0.296
Liver metastases<br>- Any vs. not present	-0.03 (-0.09 to 0.03)	0.365	-0.02 (-0.09 to 0.03)	0.417
PD-L1 TPS				
-<1%	Reference	-	Reference	-
- ≥1% and ≤49%	-0.03 (-0.08 to 0.01)	0.154	-0.03 (-0.08 to 0.01)	0.119
- ≥50%	0.04 (-0.01 to 0.10)	0.124	-0.02 (-0.10 to 0.06)	0.615
BMI (kg/m2)<br>- ≥25 vs. <25	0.01 (-0.03 to 0.04)	0.783	0.01 (-0.02 to 0.05)	0.587
Smoking habits<br>- Ever vs. never	0.03 (-0.03 to 0.10)	0.322	0.03 (-0.03 to 0.10)	0.305
Previous thoracic radiotherapy<br>- any vs. none	-0.03 (-0.10 to 0.02)	0.234	-0.04 (-0.10 to 0.01)	0.171
Autoimmune disease<br>- Yes vs. no	0.06 (-0.06 to 0.18)	0.345	0.05 (-0.06 to 0.17)	0.375

Corticosteroids [a]<br>- Yes vs. No	0.02 (-0.01 to 0.07)	0.214	0.03 (-0.01 to 0.07)	0.130
APAP [b]<br>- Yes vs. No	0.01 (-0.04 to 0.04)	0.847	0.01 (-0.03 to 0.04)	0.757
Systemic antibiotics [c]<br>- Yes vs. No	-0.01 (-0.06 to 0.04)	0.748	-0.01 (-0.06 to 0.03)	0.524
PPI<br>- Yes vs. No	-0.01 (-0.05 to 0.03)	0.612	-0.01 (-0.05 to 0.03)	0.653
Statins<br>- Yes vs. No	0.01 (-0.04 to 0.05)	0.974	-0.01 (-0.05 to 0.03)	0.692
Fibrates<br>- Yes vs. No	0.02 (-0.03 to 0.08)	0.442	0.01 (-0.04 to 0.07)	0.647
NSAIDs or ASA<br>- Yes vs. No	0.01 (-0.04 to 0.05)	0.772	-0.01 (-0.06 to 0.03)	0.683
Beta-blockers<br>- Yes vs. No	-0.01 (-0.06 to 0.03)	0.556	-0.01 (-0.05 to 0.04)	0.772
ACEi or ARBs<br>- Yes vs. No	0.01 (-0.04 to 0.04)	0.908	0.01 (-0.02 to 0.06)	0.374
Metformin<br>- Yes vs. No	0.02 (-0.02 to 0.07)	0.382	0.02 (-0.02 to 0.06)	0.373
Oral or transdermal opioids<br>- Yes vs. No	-0.01 (-0.04 to 0.03)	0.931	0.01 (-0.03 to 0.04)	0.673
Treatment setting<br>- Second vs. first line	0.04 (-0.01 to 0.10)	0.133	-0.04 (-0.13 to 0.10)	0.289
ICI<br>- Nivolumab<br>- Pembrolizumab<br>- Atezolizumab	-	-	 Reference<br>0.01 (-0.05 to 0.06)<br>0.04 (-0.04 to 0.14)	 -<br>0.864<br>0.306
Treatment type<br>- ICI only<br>- Pemetrexed-based	-	-	 Reference<br>-0.09 (-0.18 to 0.01)	 -<br>0.050

- Paclitaxel based			-0.12 (-0.24 to 0.02)	0.053

LDH, lactate dehydrogenase; log, logarithmic values; CI, confidence interval; ECOG PS, Eastern Cooperative Oncology Group Performance Status; PD-L1 TPS, programmed cell death ligand-1 tumor proportion score; BMI, body mass index; APAP, acetaminophen; PPI, proton pump inhibitors; NSAIDs, nonsteroidal anti-inflammatory drugs; ASA, acetylsalicylic acid; ACEi, angiotensin-converting enzyme inhibitors; ARBs, angiotensin II type 2 receptor blockers; ICI, immune checkpoint inihibitor.

[a] Corticosteroids indicate intake of prednisone equivalent ≥10 mg daily for at least 5 days before 14 days prior to the start of treatment (excluding premedication for chemotherapy); [b] APAP indicates a therapeutic intake of at least 1000 mg per day for more than 24 hours during the 30 days prior to the start of treatment; [c] systemic antibiotics indicate a therapeutic intake in the 30 days prior to the start of treatment.

P values derived from parametric 2-sided Wald's $\chi 2$ test with Bonferroni (α =0.01) correction for multiple comparisons. A two-sided P value of <0.05 was considered statistically significant.

Supplementary Table S3. Multivariate analysis of dNLR values by predefined clinical variables.

Covariate	dNLR values at baseline (log)		dNLR values after two cycles of treatment (log)	
	Beta (95% CI)	P value	Beta (95% CI)	P value
Age		0.708		0.534
- ≥70 years vs. <70 years	0.01 (-0.03 to 0.05)		-0.01 (-0.07 to 0.03)	
Sex		0.080		0.217
- Male vs. female	0.05 (-0.01 to 0.10)		0.03 (-0.02 to 0.10)	
ECOG PS		0.166		0.050
- 2 vs. 0 or 1	0.04 (-0.01 to 0.10)		0.07 (-0.01 to 0.14)	
Histologic subtype		0.736		0.299
- Squamous vs. nonsquamous	-0.01 (-0.07 to 0.05)		-0.03 (-0.10 to 0.03)	
Number of metastatic sites		0.277		0.780
- >2 vs. ≤2	0.03 (-0.03 to 0.10)		0.01 (-0.06 to 0.08)	
Bone metastases		0.855		0.555
- Any vs. not present	0.01 (-0.05 to 0.06)		0.02 (-0.04 to 0.08)	
Brain metastases		0.632		0.826
- Any vs. not present	-0.01 (-0.08 to 0.05)		-0.01 (-0.08 to 0.06)	
Liver metastases		0.718		0.689
- Any vs. not present	-0.01 (-0.09 to 0.06)		-0.01 (-0.10 to 0.06)	
PD-L1 TPS				
-<1%	Reference	-	Reference	-
- ≥1% and ≤49%	-0.03 (-0.09 to 0.02)	0.220	-0.04 (-0.11 to 0.01)	0.136
- ≥50%	-0.09 (-0.19 to 0.01)	0.076	-0.07 (-0.18 to 0.03)	0.181
BMI (kg/m2)		0.708		0.797
- ≥25 vs. <25	-0.01 (-0.05 to 0.04)		-0.01 (-0.06 to 0.04)	
Smoking habits		0.604		0.674
- Ever vs. never	-0.02 (-0.10 to 0.05)		0.01 (-0.06 to 0.10)	
Previous thoracic radiotherapy		0.352		0.251
- any vs. none	-0.03 (-0.10 to 0.03)		-0.04 (-0.12 to 0.03)	
Autoimmune disease		0.400		0.674
- Yes vs. no	0.06 (-0.08 to 0.21)		0.08 (-0.07 to 0.10)	

Corticosteroids [a]<br>- Yes vs. No	 0.02 (-0.02 to 0.07)	0.299	 0.03 (-0.02 to 0.09)	0.254
APAP [b]<br>- Yes vs. No	 0.01 (-0.04 to 0.05)	0.805	 0.01 (-0.04 to 0.07)	0.596
Systemic antibiotics [c]<br>- Yes vs. No	 0.01 (-0.05 to 0.07)	0.821	 -0.01 (-0.07 to 0.06)	0.983
PPI<br>- Yes vs. No	 0.01 (-0.04 to 0.06)	0.733	 0.01 (-0.05 to 0.06)	0.797
Statins<br>- Yes vs. No	 0.02 (-0.03 to 0.08)	0.426	 0.01 (-0.06 to 0.06)	0.952
Fibrates<br>- Yes vs. No	 -0.01 (-0.08 to 0.06)	0.776	 0.01 (-0.07 to 0.08)	0.939
NSAIDs or ASA<br>- Yes vs. No	 -0.04 (-0.10 to 0.01)	0.124	 -0.02 (-0.08 to 0.04)	0.517
Beta-blockers<br>- Yes vs. No	 -0.03 (-0.09 to 0.02)	0.282	 -0.02 (-0.09 to 0.04)	0.441
ACEi or ARBs<br>- Yes vs. No	 0.01 (-0.03 to 0.07)	0.464	 0.01 (-0.04 to 0.07)	0.585
Metformin<br>- Yes vs. No	 -0.01 (-0.07 to 0.04)	0.673	 -0.01 (-0.06 to 0.05)	0.870
Oral or transdermal opioids<br>- Yes vs. No	 0.02 (-0.02 to 0.07)	0.309	 0.01 (-0.04 to 0.06)	0.692
Treatment setting<br>- Second vs. first line	 -0.03 (-0.14 to 0.06)	0.483	 -0.05 (-0.16 to 0.06)	0.380
ICI<br>- Nivolumab<br>- Pembrolizumab<br>- Atezolizumab	-	-	 Reference<br>0.05 (-0.02 to 0.13)<br>-0.04 (-0.16 to 0.08)	 -<br>0.170<br>0.519
Treatment type<br>- ICI only<br>- Pemetrexed-based	-	-	 Reference<br>-0.17 (-0.29 to -0.06)	 -<br>0.020

- Paclitaxel based			-0.10 (-0.27 to 0.05)	0.184

dNLR, derived neutrophil/lymphocyte ratio; log, logarithmic values; CI, confidence interval; ECOG PS, Eastern Cooperative Oncology Group Performance Status; PD-L1 TPS, programmed cell death ligand-1 tumor proportion score; BMI, body mass index; APAP, acetaminophen; PPI, proton pump inhibitors; NSAIDs, nonsteroidal anti-inflammatory drugs; ASA, acetylsalicylic acid; ACEi, angiotensin-converting enzyme inhibitors; ARBs, angiotensin II type 2 receptor blockers; ICI, immune checkpoint inhibitor.

[a] Corticosteroids indicate intake of prednisone equivalent ≥10 mg daily for at least 5 days before 14 days prior to the start of treatment (excluding premedication for chemotherapy); [b] APAP indicates a therapeutic intake of at least 1000 mg per day for more than 24 hours during the 30 days prior to the start of treatment; [c] systemic antibiotics indicate a therapeutic intake in the 30 days prior to the start of treatment.

P values derived from parametric 2-sided Wald's $\chi 2$ test with Bonferroni (α =0.01) correction for multiple comparisons. A two-sided P value of <0.05 was considered statistically significant.

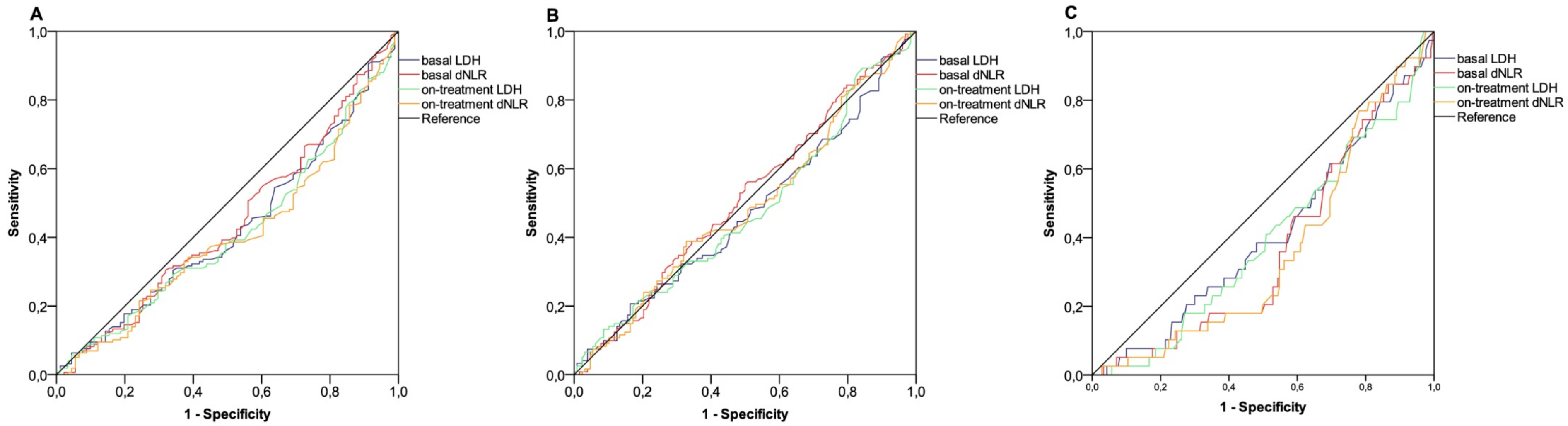

Supplementary Figure S1. ROC curve analysis.

(A) General population: ROC curve analysis showing the performance of LDH levels and dNLR values assessed at baseline (basal) and after two cycle of PD-(L)1 blockade therapy (on-treatment) in predicting the occurrence of any grade irAEs; AUC relative value: basal LDH 0.427 (95% CI 0.354-0.499), *P*=0.054; on-treatment LDH 0.414 (95% CI 0.342-0.486), *P*=0.024; basal dNLR 0.446 (95% CI 0.371-0.520), *P*=0.153; on-treatment dNLR 0.405 (95% CI 0.333-0.447), *P*=0.013. (B) First-line therapy population: ROC curve analysis showing the performance of LDH levels and dNLR values assessed at baseline (basal) and after two cycle of PD-(L)1 blockade therapy (on-treatment) in predicting the occurrence of grades 1-2 irAEs; AUC relative value: basal LDH 0.473 (95% CI 0.401-0.545), *P*=0.456; on-treatment LDH 0.477 (95% CI 0.405-0.549), *P*=0.528; basal dNLR 0.513 (95% CI 0.441-0.585), *P*=0.720; on-treatment dNLR 0.488 (95% CI 0.415-0.530), *P*=0.735. (C) Second-line therapy population: ROC curve analysis showing the performance of LDH levels and dNLR values assessed at baseline (basal) and after two cycle of PD-(L)1 blockade therapy (on-treatment) in predicting the occurrence of grades 3-4 irAEs; AUC relative value: basal LDH 0.409 (95% CI 0.313-0.505), *P*=0.071; on-treatment LDH 0.394 (95% CI 0.302-0.487), *P*=0.036; basal dNLR 0.371 (95% CI 0.282-0.459), *P*=0.010; on-treatment dNLR 0.363 (95% CI 0.268-0.449), *P*=0.007.
ROC, receiver operating characteristic; LDH, lactate dehydrogenase; dNLR, derived neutrophil/lymphocyte ratio; PD-(L)1, programmed cell death (ligand)-1; irAEs, immune-related adverse events; AUC, area under the curve; CI, confidence interval.
